# Toward Flexible Surface‐Enhanced Raman Scattering (SERS) Sensors for Point‐of‐Care Diagnostics

**DOI:** 10.1002/advs.201900925

**Published:** 2019-07-02

**Authors:** Kaichen Xu, Rui Zhou, Kuniharu Takei, Minghui Hong

**Affiliations:** ^1^ Department of Electrical and Computer Engineering National University of Singapore 4 Engineering Drive 3 Singapore 117576 Singapore; ^2^ Department of Physics and Electronics Osaka Prefecture University Sakai Osaka 599‐8531 Japan; ^3^ School of Aerospace Engineering Xiamen University 422 Siming South Road, Siming District Xiamen Fujian 361005 P. R. China

**Keywords:** flexible sensors, in situ detection, surface‐enhanced Raman scattering, swab‐sampling approach, tunable surface plasmon resonance

## Abstract

Surface‐enhanced Raman scattering (SERS) spectroscopy provides a noninvasive and highly sensitive route for fingerprint and label‐free detection of a wide range of molecules. Recently, flexible SERS has attracted increasingly tremendous research interest due to its unique advantages compared to rigid substrate‐based SERS. Here, the latest advances in flexible substrate‐based SERS diagnostic devices are investigated in‐depth. First, the intriguing prospect of point‐of‐care diagnostics is briefly described, followed by an introduction to the cutting‐edge SERS technique. Then, the focus is moved from conventional rigid substrate‐based SERS to the emerging flexible SERS technique. The main part of this report highlights the recent three categories of flexible SERS substrates, including actively tunable SERS, swab‐sampling strategy, and the in situ SERS detection approach. Furthermore, other promising means of flexible SERS are also introduced. The flexible SERS substrates with low‐cost, batch‐fabrication, and easy‐to‐operate characteristics can be integrated into portable Raman spectroscopes for point‐of‐care diagnostics, which are conceivable to penetrate global markets and households as next‐generation wearable sensors in the near future.

## 1 Introduction

The past several decades have witnessed tremendous advances in nanotechnology, smartphone technologies, and wireless communication, which ceaselessly propel the development of point‐of‐care (POC) diagnostics in diverse fields, including healthcare, food safety, environment, medicine, and biology.^1^ Different from the conventional sophisticated laboratory‐based testing generally requiring complex laboratory instruments, multiple preparation steps, and well‐skilled technicians, the on‐demand, onsite, and easy‐to‐use POC diagnostics target at precisely providing rapid detection and response at the nonlaboratory environment, which is capable of achieving real‐time measurements, rather than historical values for monitoring health status or controlling infectious diseases.^2^ Such POC testing routes are particularly appealing in remote and resource‐limited areas, where operation of advanced instruments by uninterrupted electricity and water in desired environmental conditions is not available. In this case, battery‐powered and even self‐powered portable devices are favorable to easy transport and able to perform data collection, uploading, and analysis/interpretation in a fast manner through integration with smartphone‐based wireless systems.^3^ As the headstream of POC diagnostics, the sensing modules with enhanced physical, chemical, optical, and electrical properties play a vital role.^4^ They are usually tailored to POC applications in terms of massive cost‐efficient production capability, reproducible measurements from batch to batch, free from cold requirement for storage/shipping, biocompatibility with bodily fluids detection, and multiplexed on‐chip measurement without tedious pre‐ or postsample preparation steps.^5^ A typical example of POC monitoring is single‐use glucose lateral‐flow test strips based on immunoassay, which are prevalently applied for personal diabetes evaluation at home due to the easy‐to‐operate characteristics with minimum training.^6^ However, a positive/negative qualitative answer is usually provided by such traditional lateral‐flow assays. Furthermore, microfluidic chips coupled with array‐based sensors have been extensively studied, taking advantage of the parallel multiple microchannels to capture, isolate, and quantitatively analyze diverse biosamples (e.g., cells, proteins, and nuclei acids) on lab‐chip systems for POC measurement.^7^, ^8^ For the general topic of POC testing, interested readers may refer to the excellent review by Nayak et al.^9^

Another promising category is flexible wearable devices, such as thermal, electrical, chemical and optical sensors, etc.^10^, ^11^, ^12^, ^13^, ^14^, ^15^ The rapid, easy‐to‐operate and cost‐effective characteristics of the flexible sensors render them timely monitoring, care or management in nonlaboratory settings.^16^, ^17^, ^18^, ^19^ Because of these merits, recent remarkable efforts have been focused on enhancing the performance of flexible sensors in terms of bendability,^20^, ^21^, ^22^ stretchability,^23^, ^24^, ^25^ ultrasensitivity,^26^, ^27^, ^28^ transparency,^29^, ^30^, ^31^ and multifunctionality.^32^ Among a variety of flexible biosensors, surface‐enhanced Raman scattering (SERS) has attracted enormous research attentions because of its label‐free and fingerprint detection capability to noninvasively trace extremely low concentration analytes.^33^, ^34^, ^35^, ^36^, ^37^, ^38^, ^39^ In particular, the SERS technique building from flexible materials provides superior advantages over conventional SERS detection means based on rigid substrates.^40^, ^41^, ^42^, ^43^, ^44^ For instance, in the application of environmental monitoring, the general approach for conventional rigid substrate‐based SERS detection requires the complicated extraction of analytes from out‐of‐door objects of interest and tedious sample preparation steps in the laboratory settings before the adsorption of analytes on the SERS substrate for analyses, which is not suitable for practical applications. In contrast, the emerging flexible SERS technique relies on the backbone materials with soft, flexible, and optically transparent features to be intimately contact with the arbitrary surfaces for in situ and onsite detection.^40^, ^42^, ^45^ Furthermore, the development of handheld portable Raman spectrometer coupled with such flexible SERS nanotechnologies render the promising in field identification of bio‐chemicals for POC diagnostics, although there is a couple of challenges to overcome, such as relatively low sensitivity, batch‐to‐batch consistency, macromolecules' identification, multiplexed analyses as well as costly portable Raman spectrometers.^46^ A number of research efforts are thus involved in breaking through these obstacles to realize the POC diagnostics to meet the increasing demands for a wide range of applications, especially in resource‐limited environment.^5^, ^40^, ^47^, ^48^

It is well known that, since its discovery, tremendous efforts have been devoted to exploiting versatile SERS substrates building from rigid materials (silicon, glass and metal sheet, etc.) with abundant sub‐10 nm gap structures based on chemical syntheses or advanced lithographic routes to allow the possible identification of single molecule.^33^, ^49^, ^50^, ^51^, ^52^, ^53^, ^54^, ^55^, ^56^, ^57^ Generally, a superior SERS‐active substrate requires high‐density hot spots over a large area with good uniformity, signal reproducibility, high enhancement factors and low fabrication cost. However, these rigid material–based SERS platforms have some drawbacks when performing the real‐world applications. Thus, current research priority is being extended to flexible material–based SERS devices with the distinct superiorities that the traditional rigid SERS substrates are not accessible to. Until now, numerous materials have been applied in flexible SERS substrates as the building blocks, including flexible polymers,^58^, ^59^, ^60^, ^61^, ^62^, ^63^ papers,^64^, ^65^, ^66^, ^67^, ^68^ graphene/graphene oxide,^69^, ^70^, ^71^, ^72^, ^73^ and nanowires,^43^, ^74^ etc. The properties of these flexible materials determine the functionalities of the flexible SERS applications. For example, taking advantage of the stretchability of polydimethyl‐siloxane (PDMS) membrane, Kang et al. revealed the universal relationships between plasmon resonance and SERS, providing the theoretic guidance to engineer on‐demand high‐performance SERS devices.^75^ Featuring its high stretchability to actively control nanogaps, Mitomo et al. realized the highly sensitive detection of bio‐macromolecules by SERS based on the elastomeric material.^76^ Furthermore, because of the favorable traits of the paper, such as flexibility, conformability and hierarchical vasculature, Lee et al. initiated a new venue by using the SERS swab for rapid analytes' collection from complex real‐world surfaces.^77^ These research work has endowed the flexible SERS technique with unprecedented functionalities to meet the increasingly on‐demand requirement in numerous applications, such as the pesticides' in situ detection on various fruit surfaces,^78^ onsite diagnostics of aquatic products,^79^ noninvasive monitoring of the glucose or uric acid in human tears^65^ as well as wearable cryptographic technologies.^80^

**Figure 1:**
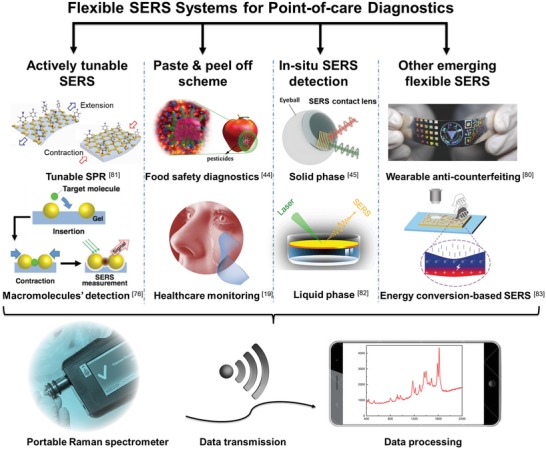
Schematic illustration of current various types of flexible SERS platforms integrated with a portable Raman spectrometer and smartphone for point‐of‐care diagnostics. Reproduced with permission.^81^ Copyright 2018, Royal Society of Chemistry. Reproduced with permission.^76^ Copyright 2016, John Wiley & Sons. Reproduced with permission.^44^ Copyright 2017, American Chemical Society. Reproduced with permission.^45^ Copyright 2016, Wiley‐VCH. Reproduced with permission.^82^ Copyright 2016, Springer Nature. Reproduced with permission.^80^ Copyright 2016, Wiley‐VCH. Reproduced with permission.^83^ Copyright 2017, John Wiley & Sons.

Here, we present a comprehensive overview of recent advances in this emerging field of flexible SERS techniques. In particular, based on the multifunctional purposes that the flexible SERS aims to achieve, this report mainly demonstrates three categories of current flexible SERS platforms: i) actively tunable SERS, ii) swab‐sampling SERS strategy, and iii) in situ SERS detection approach as illustrated in **Figure** **1**. These flexible SERS platforms coupled with portable Raman spectrometers provide intriguing applications toward POC diagnostics. The major challenges in these three sub‐fields are highlighted, such as the macromolecules' identification, poor adhesion between the active plasmonic nanostructures and flexible substrates, low analytes' extraction efficiency during the swab‐sampling as well as large thickness of the flexible substrate for in situ detection. In addition, other emerging flexible SERS devices are also introduced, including the energy conversion based flexible SERS, microsphere‐enhanced Raman spectroscope and wearable anticounterfeiting devices. Finally, the future trends to transform the flexible SERS technique into real‐world applications for POC testing are provided. We believe this report can provide a timely reference for the researchers with the guidance to develop high‐quality flexible SERS platforms to further satisfy the booming POC diagnostics.

## 2 Actively Tunable SERS

Although lots of advances have been made in the SERS area since its discovery, most of the SERS substrates using rigid materials as the building blocks have weak optical tunability, which exhibits remarkably strong SERS signals for one excitation wavelength, but poor enhancement for another incident laser wavelength. Meanwhile, the majority of research focuses on the exploration of hybrid nanostructures with the interparticle feature size as small as possible to achieve giant local electric field enhancement. However, such tiny nanogaps are not always advantageous for the sensitive identification of large biomolecules, such as proteins, bacteria and other macromolecular biomarkers. The recently emerging flexible and stretchable SERS devices allow possibilities to address the aforementioned issues.

### 2.1 Judiciously Tuning Surface Plasmon Resonance Based on Stretchable SERS‐Active Substrates

**Figure 2:**
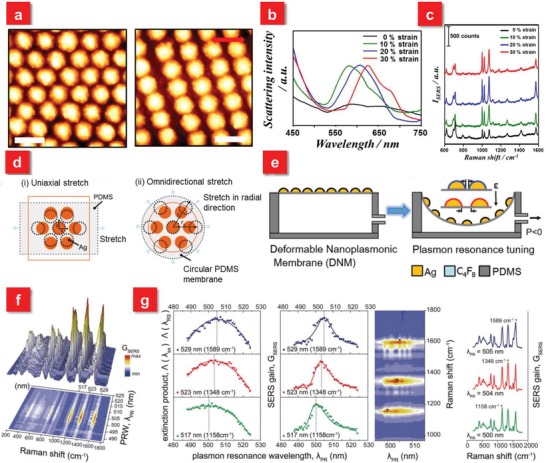
a) AFM images of the plasmonic cap arrays under applied strains of 0 (left) and 30% (right). The red double sided arrow denotes the strain direction. Scale bar: 500 nm. b) Scattering spectra of the plasmonic cap arrays at different applied strains. c) Tunable SERS spectra of the 1 × 10^−6^ m benzenethiol‐adsorbed plasmonic cap arrays as a function of the applied strain. Reproduced with permission.^101^ Copyright 2013, American Chemical Society. d) Schematic illustration of interstitial gap modulation for i) uniaxial stretching and ii) omnidirectional stretching. e) Deformable nanoplasmonic membranes (DNMs) to allow large extensibility for continuously increasing the gap distance between neighboring nanoislands in tuning plasmon resonance. f) A quantitative investigation of plasmon resonance and individual SERS peaks. g) The analytic correlation between plasmon resonance and individual SERS peaks. The individual SERS peak has the maximum gain at a specific plasmon resonance wavelength, which has the maximum extinction product of an excitation and the corresponding Raman scattering wavelengths (left). Consequently, the relative SERS peak heights substantially vary depending on the plasmon resonance of SERS probes (right). Reproduced with permission.^75^ Copyright 2014, John Wiley & Sons.

Generally, the primary contribution to SERS performance is dominated by the electromagnetic field enhancement, where excitation light and Raman emission signals have strong interactions with the plasmon resonance. Notably, for the theoretical mechanisms of SERS, interested readers may refer to the excellent reviews by Lane et al. and Ding et al.^84^, ^85^ It has been widely reported that the peak position of plasmon resonance with respect to excitation and Raman shift wavelengths determines the SERS enhancement for a plasmonic system. However, most traditional SERS devices based on rigid substrates fabricated by the top‐down approach, bottom‐up assembly or template‐assisted fabrication have a fixed surface plasmon resonance (SPR) position or limited SPR tunability after the preparation.^86^, ^87^, ^88^ To achieve the maximum SERS effect, a judicious design is typically required for a specific optical range in the SPR spectra before the fabrication, which is not beneficial for practical applications, especially in the real‐time detection of biomolecules with extremely low concentrations. Recently, the rising tunable SERS systems can resolve aforementioned drawbacks because of their capability to actively control SPR.^89^ Several strategies to dynamically modulate SPR usually rely on external electrical,^90^ magnetic,^91^ thermal,^92^, ^93^ or light stimulus^94^ etc. to tune the plasmon resonance peak, which, however has restriction in on‐demand SERS applications. Fortunately, the flexible and stretchable materials, such as gels and elastomers, act as superior backbones to actively change SPR through tuning surface interparticle gap distance based on applied strain, rendering promising applications in controlling SERS signals.^95^, ^96^, ^97^, ^98^, ^99^, ^100^ For example, Kang et al. demonstrated active plasmonic cap arrays on the flexible PDMS substrate by colloidal lithography coupled with lift‐off soft lithography, providing real‐time optical tunability for reliable SERS sensors. Via applying the uniaxial strain on the elastomeric substrate, the intercaps gap distance tends to be larger along the applied strain, while the dimension between adjacent plasmonic caps becomes closer in the direction perpendicular to the applied strain (**Figure** **2**a). Such phenomena give rise to a tunable and reversible plasmonic extinction ranging from 581 to 625 nm because of the plasmonic coupling effect (Figure 2b). It is found that under the 20% applied strain, the SERS performance at the laser excitation wavelength of 632.8 nm is maximized for benzenethiol detection due to the optimized combined effects of the SPR position as well as the re‐arrangement of the plamsonic caps (Figure 2c).^101^ Furthermore, 2D materials, such as graphene or MoS_2_, are also good candidates to be integrated with metallic nanoparticles as well as flexible substrates to enhance SERS signals.^70^, ^102^ To overcome the low stretchability of the 2D materials, it is demonstrated by Chen et al. that the wrinkled graphene/Au nanoparticles hybrid platform can be a stretchable substrate for sensitive and stable SERS applications.^70^ With increasing tensile strain from 0 to 30%, the resonance peak demonstrates a blueshift from ≈800 to ≈750 nm, approaching the wavelength of excited laser 633 nm. The highest Raman intensity of mode 612 cm^−1^ for Rhodamine 6G molecules (concentration: 10^−6^ m) is achieved at the uniaxial tensile strain of 10%. The further increase of tensile strain decreases the number of absorbed R6G molecules per unit area in the range of laser detection spot size, but a similar enhancement factor (EF) to the initial value could be preserved even under a tensile strain of 50%. Such wrinkles in graphene not only provide more hot spots for SERS enhancement, but also enable the graphene to endure larger deformation. The SERS substrate exhibits good reproducibility of the enhancement performance after 100 stretching cycles under 30% tensile strain.

The aforementioned work employs the uniaxial stretching strategy, an easier controllable method, to modulate plasmon resonance. Nevertheless, this approach is not appropriate to get the clearly quantitative relationship between the excitation wavelength and SERS signals with respect to plasmon resonance. The reason is that it suffers from the issue of polarization sensitivity because the plasmon resonance is blue‐shifted for one direction parallel to the applied strain, but becomes red‐shifted for the other orientation due to the contraction in the perpendicular direction as shown in Figure 2d.^81^, ^101^, ^103^, ^104^ To overcome this weakness, the omnidirectional stretch is applied, leading to the monotonic and continuous tuning of plasmon resonance (Figure 2e).^75^ A deformable nanoplasmonic membrane (DNM) consisting of a thin and circular PDMS membrane with size controlled silver nanoislands enables the omnidirectional and uniform elongation through varying air pressure, which demonstrates a large tuning of the plasmon resonance over the Stokes' Raman shift range with a tuning resolution of 1 nm due to the reversible interparticle gap distance change (Figure 2f). Relying on this capability, it reveals that the individual SERS peak has the maximum gains at the maximum products of extinction values at an excitation and the corresponding Raman scattering wavelengths (Figure 2g). Meanwhile, such omnidirectionally stretchable strategy is also extended to other promising research fields, such as supercapacitors,^105^ pressure sensors,^106^ photodetectors,^107^ and other flexible devices.^108^

### 2.2 Macromolecules' Diagnostics Based on Stretchable SERS

In addition to the tuning of plasmon resonance to a specific wavelength based on the stretchable elastomeric platforms, controllable gap distance over flexible substrates is also favorable for label‐free detection of certain analytes, especially larger biomolecules, such as protein and bacteria.^47^, ^76^ In the past decades, tremendous efforts have been devoted to developing extremely small interparticle nanogaps even down to ≈5 nm with a high‐packing density by the rational chemical reactions^109^, ^110^, ^111^, ^112^, ^113^ or state‐of‐art lithographic methods.^49^, ^114^, ^115^ Such ultrasmall plasmonic nanogaps resemble nanocavities to strongly focus the incident light, resulting in giant localized electromagnetic field, even under the off‐resonant conditions, which can detect analytes of interest at the single molecule level. However, it has been found that the highest‐packing density of plasmonic superlattice does not always demonstrate the most efficient SERS performance, which indicates that “more is not always better” for SERS applications.^116^ Particularly, in the biomedical applications, the size of biological and chemical species is much larger than typical chemical compounds, such as dyes, drugs, pesticides or explosives. These large analytes, such as bacteria or proteins, usually suspend on the conventional SERS substrates' surface, which exhibits poor accessibility into the ultrasmall nanogaps.^117^

**Figure 3:**
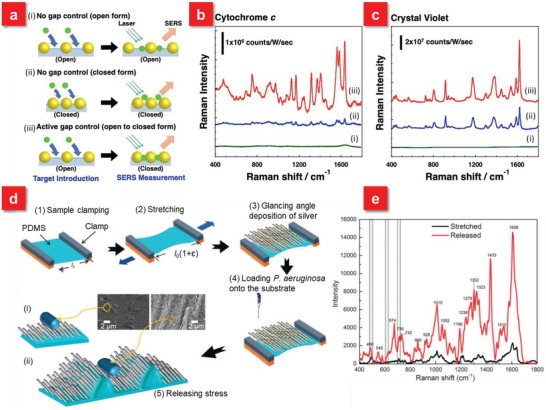
a) Schematic illustrations of three approaches: i) no gap control as “open form”—the approach where analytes were injected onto a gel in an expanded state (in MilliQ water) and analyzed as it is; ii) no gap control as “closed form”—the approach where analytes were injected onto a gel in a contracted state (in 1 m NaCl solution) and analyzed as it is; and iii) active gap control as “open‐to‐closed form”—the approach that target molecules were injected onto a gel is an expanded state (in MilliQ water) and analyzed after contraction (in 1 m NaCl solution). b) SERS spectra of cytochrome *c* and c) crystal violet for each approach. The concentrations of the analytes used in these experiments were 0.1 mg mL^−1^ for cytochrome *c* and 1 × 10^−6^ m for crystal violet. SERS measurements were performed using 532 nm laser excitation (1.5 mW for cytochrome *c* and 75 µW for crystal violet) with a 20 × objective lens. Data acquisition time is 10 s. The cumulative number is 10 at each position. Background correction was performed by the subtraction of a fitting curve as a quadratic function. Reproduced with permission.^76^ Copyright 2016, John Wiley & Sons. d) A schematic illustration of the fabrication of the AgNR‐PDMS substrate. Schematic showing *P. aeruginosa* on i) prestretched and ii) wrinkled AgNR‐PDMS substrates and corresponding SEM of *P. aeruginosa* on prestretched and wrinkled AgNR‐PDMS substrates. e) SERS spectra of *P. aeruginosa* on prestretched and relaxed AgNR‐PDMS substrates. Reproduced with permission.^47^ Copyright 2015, Royal Society of Chemistry.

To address these issues, Mitomo et al. proposed an open‐to‐closed SERS system building from the stretchable polyacrylic acid gel–based substrate, providing the active gap distance control for the sensitive identification of bio‐macromolecules (**Figure** **3**a).^76^ When the nanogaps are opened continuously, no Raman signals from Cytochrome *c* (a protein), can be detected at the laser excitation wavelength of 532 nm. As the nanogaps are continuously closed, very weak SERS signals are obtained because of the enhanced electric field. Significantly, when the molecules are first injected into the opened gaps, which are then closed for the following detection, it is found that the intensity of SERS signals exhibits around ten times larger than the continuously closed SERS system (Figure 3b). For comparison, when crystal violet (a well‐known Raman‐active molecule) is applied for detection, the increase of SERS signals is only about twofold (Figure 3c). When the size of substrate gel is changed from 1 (original size) to 3 in length, the average gap distance could be increased from 0 to about 40 nm, which is much larger than the size of proteins, indicating that the gap change is good enough for protein insertion and detection. However, crystal violet is a small molecule, which can be attached or inserted into a hot spot, even if the gap size is short. Thus, in the case of crystal violet, the effect of active gap control is small (only approximately twofold enhancement). This indicates that the tunable open‐to‐closed SERS system can improve the insertion efficiency of the target molecules, especially the larger proteins, into nanogaps based on actively controlling gap distance. Another strategy to enhance the SERS signals of large analytes is to couple the macrospecies into 3D structures. For instance, a promising prestretch and release scheme is demonstrated to identify bacteria with the detection limit down to 10^4^ CFU mL^−1^.^47^ The Ag nanorods (AgNR) are first fabricated on the prestretched PDMS by oblique angle deposition, which is then directly pipetted with bacterial suspension. The stress is released for SERS detection (Figure 3d).^47^, ^118^ It is found that the buckled AgNR‐PDMS substrates exhibit about eleven‐fold enhancement at the laser excitation wavelength of 514 nm in comparison with that of the prestretched AgNR‐PDMS film as demonstrated in Figure 3e. Such difference is primarily attributed to the formation of grooves after the release of the strain on PDMS, which boosts the effective contact area of the nanorods to bacteria. Hence, the flexible and stretchable SERS systems can not only afford reversible plasmon resonance shift but also provide a significant route for highly sensitive detection of bio‐macromolecules through actively modulating the interparticle distance. The tunable properties of these flexible SERS substrates have unprecedented opportunities to be applied in on‐demand sites to maximize the Raman signals.

## 3 Paste and Peel off Scheme (Swab‐Sampling Approach) for SERS Detection

The swab sampling approach (also called “paste and peel off scheme”) is an efficient strategy to detect organic and inorganic contaminants, such as pesticides, dusts, metals, spray drift, or residues, on numerous surfaces, which has been applied in a wide range of fields. For example, the swab sampling is used for cleaning validation to determine residual active pharmaceutical ingredient levels in samples collected after cleaning equipment.^119^ Furthermore, this approach is also extensively employed for passenger screening at airports in combination with ion mobility spectroscopy. Therefore, it is conceivable that the swab sampling strategy coupled with SERS techniques affords a promising route toward POC diagnostics.

Nevertheless, most conventional SERS analyses are based on rigid supports, such as silicon, glass sheet, and aluminum foil, which require complex samples pretreatment. The typical procedure is to extract the objective analytes to be dissolved in the suitable solvent and then be adsorbed onto the as‐designed plasmonic templates for SERS analyses. Such tedious sample pretreatment processes hinder the practical nonlaboratory settings' monitoring. Furthermore, the efficiency of analytes' collection is also a crucial factor when the concentration of unknown species is extremely low. In order to satisfy the requirement of increasingly demanded POC diagnostics for onsite screening applications, it is essential to develop a universal sample collection approach with ease and high efficiency. The emerging flexible substrate materials, such as paper, polymer carbon, nanofiber or metal foam/net, are recently demonstrated to support the plasmonic nanostructures.^120^, ^121^, ^122^, ^123^, ^124^, ^125^, ^126^, ^127^ Featuring their most remarkable property of flexibility, the unique SERS substrates building from these flexible materials can be swabbed onto real‐world surfaces with arbitrary morphology for objective analytes' collection.

### 3.1 Paper‐Based Swab Sampling

**Figure 4:**
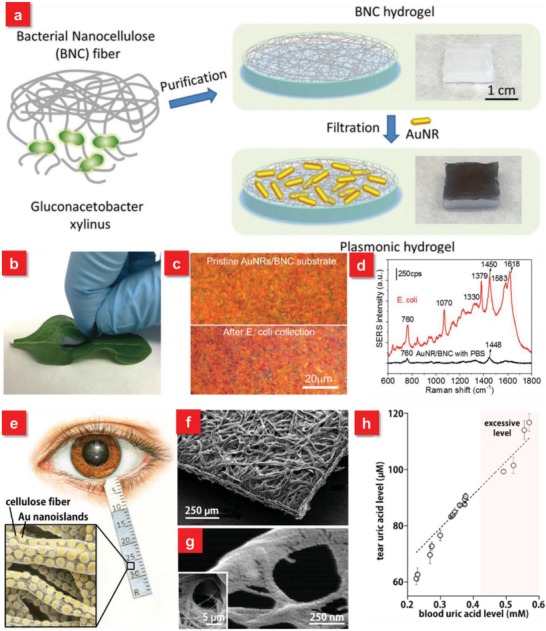
a) Schematic illustration showing the steps involved in the preparation of plasmonic hydrogel from bacterial nanocellulose and photographs showing BNC hydrogel and plasmonic BNC hydrogel. b) Photograph showing a plasmonic BNC SERS substrate being swabbed on a spinach leaf surface intentionally contaminated with *E. coli*. c) Bright field optical images of pristine AuNRs/BNC paper and *E. coli* adsorbed on AuNRs/BNC paper collected by swabbing. d) Representative SERS spectra collected from *E. coli* adsorbed on the AuNRs/BNC swab showing characteristic Raman bands of *E. coli*. Reproduced with permission.^128^ Copyright 2016, John Wiley & Sons. e) Schematic diagram of a plasmonic Schirmer strip for SERS‐based tear screening. f) A plasmonic Schirmer strip and g) cellulose nanofibers covered with Au nanoislands in Volmer−Weber mode. h) Correlation between uric acid level in tears and in blood. Reproduced with permission.^65^ Copyright 2016, American Chemical Society.

Among a wide range of flexible materials, paper is attracting increased research attention, not only in SERS applications, but also in catalysis, energy harvesting and storage because of its diverse advantages, such as large surface area, excellent wicking characteristic, compatibility, biodegradability as well as low cost.^65^, ^66^, ^72^, ^77^, ^128^, ^129^, ^130^, ^131^, ^132^, ^133^, ^134^, ^135^ Lee et al. demonstrated the first SERS swab approach for the rapid detection of analytes from complex real‐world samples based on common filter paper decorated with gold nanorods.^77^ Furthermore, in order to concentrate the analytes within the detection volume to improve the sensitivity, a paper‐based SERS substrate with the functionality of both surface swab and lateral‐flow dipstick is reported.^129^ Via making use of the capillary‐action wicking of cellulose, the inkjet‐printed flexible SERS substrate can reach the detection limit as low as 95 fg of R6G, comparable to the values reported for sophisticated lithography methods. To further boost the EF, a cost‐effect filter paper integrated with multibranched gold nanoantennas (WGN) is reported for the identification of biomolecular species, which gives rise to the EF of ≈10^9^ with the lowest concentration of 100 × 10^−12^ m due to the intense electromagnetic fields on the sharp tips of protrusions.^130^ The wetted WGN‐paper can be swabbed across the apple surface contaminated with methyl pharthion (26.3 µg), a toxic, organophosphorus chemical agent applied as a pesticide on fruits for SERS analysis. In addition, sandpaper with µm scale roughness is also applied for swab sampling test of triazophos on different surfaces.^131^ In particular, among the different kinds of papers composed of cellulosic materials, dependent on processing methods and the material source, bacterial nanocellulose (BNC) is a special one because of its 3D nanofibrous structure (**Figure** **4**a). Tian et al. reported a nanocellulose‐based substrate adsorbed with gold nanorods for the efficient collection, recognition and detection of Escherichia coli (E. coli) on the surface of a spinach leaf (Figure 4b–d).^128^ Different from the conventional filter paper with microcellulose fibrous, the BNC film is endowed with the cellulose nanofibril network with larger surface area to form plasmonic nanostructures. In addition, paper‐based SERS substrates also play a significant role in daily healthcare monitoring, such as the identification of biomarkers in human tears. Relying on the hygroscopic nature of cellulose fibers, a plasmonic Schirmer strip was recently reported for rapid bioassays of tear molecules by inserting a paper strip into the lower eyelid of the human eyes to efficiently collect human tears, which exhibits enormous potentials in monitoring diverse biomarkers such as glucose, uric acid, or ascorbic acid (Figure 4e–h).^65^ These paper‐based flexible SERS substrates with inexpensive, easy‐to‐use and highly sensitive features afford immediate applications for onsite detection of a wide range of analytes.

### 3.2 Performance‐Enhancement of SERS Swab Sampling

**Figure 5:**
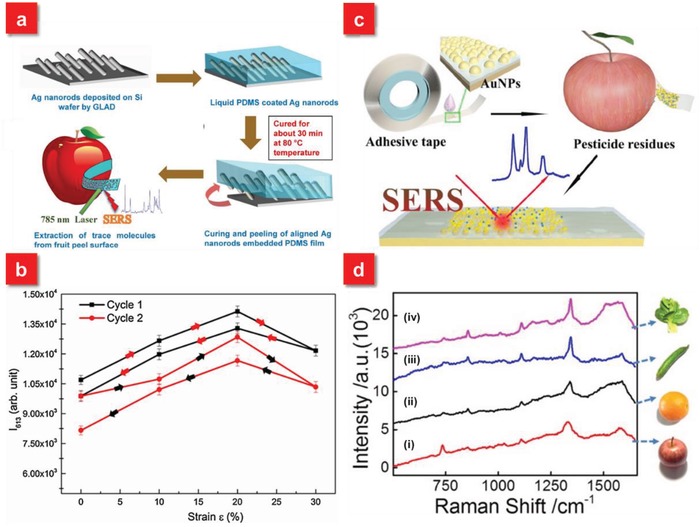
a) A schematic illustration of the fabrication of AgNRs embedded PDMS SERS substrates. b) The SERS response of AgNRs arrays on flexible PDMS substrate as a function of tensile strain (ε). Reproduced with permission.^138^ Copyright 2016, Elsevier B.V. c) Schematic illustration of the fabrication of SERS tape and extraction of targets from fruit peel surface (apple) for SERS analysis. d) Raman spectra of parathion‐methyl extracted from the surfaces of i) apples, ii) oranges, iii) cucumbers, and iv) green vegetables using SERS tape. Reproduced with permission.^41^ Copyright 2016, American Chemical Society.

Although the swab sampling test is a rapid and efficient approach for POC diagnostics, several challenges should be carefully considered and solved. For example, there is a portion of plasmonic nanostructures lost during the collection of molecules from the complex surfaces due to the low adhesion of metallic nanoparticles on the supporting flexible substrates. This may lead to the degradation of SERS substrates' sensitivity as well as contamination of costly and rare objects, such as antique or famous painting.^136^ Meanwhile, the robustness of the flexible SERS substrates against external forces is also vital, which can retain the SERS activity during the mechanical deformation.^137^ Hence, it is of high significance to develop the flexible SERS substrates with strong adhesion between the plasmonic nanostructures and beneath supporting layer as well as mechanically robust properties. To realize such a promising target, Kumar et al. demonstrated a robust, flexible and large‐area SERS substrates via embedding Ag nanorods into the PDMS polymer.^138^ First, a glancing angle deposition method is applied to prepare 3D well‐aligned silver nanorods (AgNRs) array on a rigid substrate. The PDMS solution is then poured, cured and peeled off from the AgNRs decorated rigid surfaces, allowing the realization of free standing and flexible AgNRs embedded flexible SERS substrate (**Figure** **5**a). A scotch‐tape peel test after repeating three times shows no apparent impairment of the structures on the tape surface and the ultrasonication of the product for 1 h also exhibits the AgNRs arrays films strongly adhered to surface due to the mechanical interlocking effect.^139^, ^140^ Furthermore, the AgNRs embedded PDMS substrates can retain excellent SERS performance after the mechanical deformation, which can preserve 90% of its initial SERS intensity (Figure 5b). These superior performances of the AgNRs based PDMS flexible SERS substrates enable the detection limit of Thiram swabbed from fruit peel down to ≈10^−9^.

Generally, the strategy of SERS swab sampling is required to apply the organic solvent, such as ethanol, to wet the objective surfaces or flexible substrates for the collection of analytes. In order to further enhance the extraction efficiency of molecules with high speed from complex surfaces, a flexible and adhesive SERS film is demonstrated with an adhesive tape as the backbone material.^41^, ^141^ Different from the traditional flexible SERS substrates, the “SERS tape” not only exhibits the flexible property to allow the intimate contact with arbitrary surfaces, but also provides the “sticky” feature from adhesive tape as illustrated in Figure 5c. Such unique sticky characteristic affords highly efficient extraction of analytes directly from the targeted surfaces without the assistance of any organic solution and furthermore improves the adhesion of plasmonic nanoparticles on the flexible substrate, preserving high SERS activity during the swab sampling of analytes. Based on the “paste and peel off” scheme, the flexible SERS tape presents much higher sampling efficiency of pesticide residues on the fruit surfaces in comparison with other work.^132^

**Figure 6:**
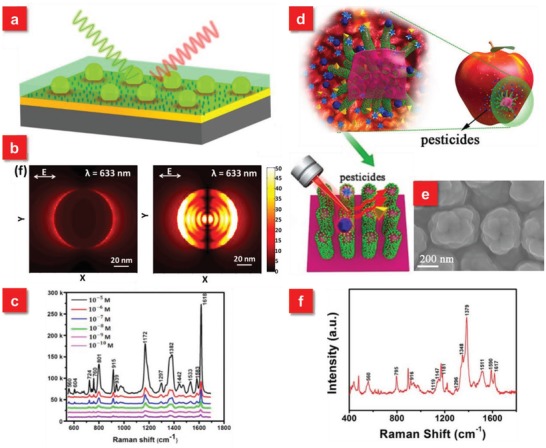
a) Schematic illustration of particle‐on‐film configuration for SERS detection. b) E‐field profiles in the *XY* plane of single Au nanoparticle on SiO_2_ substrate (left) and particle‐on‐film configuration (right). c) Detection limit measurements of crystal violet molecules by the particle‐on‐film substrates. Reproduced with permission.^142^ Copyright 2017, American Chemical Society. d) Schematic illustration of the high‐performance G‐SERS substrate via a “press and peel‐off” approach. e) SEM image of the G‐SERS substrate after depositing with Ag. f) SERS spectra of multiple components of pesticide residues (TMTD, MG, MPT) on cucumber peels using the G‐SERS substrate. Reproduced with permission.^44^ Copyright 2017, American Chemical Society.

Most reported swabbing SERS substrates are 2D planar systems, where a number of hot spots exist in the one Cartesian *x*–*y* plane.^41^, ^120^, ^142^, ^143^ To enhance the SERS performance of such 2D systems, a particle‐on‐film structure based on a sandwich configuration of particle/molecule/film is a promising strategy, which exhibits highly sensitive SERS detection (down to 10^−10^ m) because of the formation of numerous hot spots at the particle‐film interface, whereas an individual gel tape or Au film shows low or no SERS activity (**Figure** **6**a–c).^142^ Furthermore, in order to increase the density of hot spots, especially in the vertical dimension, 3D SERS swabbing systems based on flexible materials are also demonstrated.^44^, ^60^, ^123^, ^144^ One distinct advantage for the 3D flexible SERS substrate is that the 3D morphology with large aspect ratios can enhance the adsorption volume and accessibility of analytes from the complex real‐world surfaces during the swab sampling of analytes, which are possible to further enhance the sensitivity. Inspired by gecko, a talented wall walker because of the large contact area from high density of nanoscale tentacles on their toe‐pads, Wang et al. demonstrated a gecko‐like flexible SERS (G‐SERS) platform via depositing Ag nanoparticles on PDMS nanotentacles array (Figure 6d,e).^44^ Such G‐SERS system not only exhibits prominent SERS activity (EF ≈ 10^7^) with superior reproducibility (RSD ≈ 5.8%), but also provides countless nanoscale “tentacles” (≈10^8^ cm^−2^) with high flexibility. Ascribed to the strong adhesive force as well as large contact area toward topological surfaces, the “tentacles” array can freely access the microarea, leading to the highly efficient extraction of molecules from real samples of interest via a “press and peel‐off” approach, which enables the direct sampling and sensitive detection of pesticide on cucumber (10 ng cm^−2^), apple (1.6 ng cm^−2^), grape (10 ng cm^−2^), as well as multiple analytes (Figure 6f). Furthermore, a large‐scale flexible film composed of Ag nanoparticles decorated polyacrylonitrile (PAN) nanohumps array was introduced by Li et al.^60^ Via optimizing the sputtering duration and bending the film during the Ag deposition, the 3D flexible SERS substrates with high‐density hot spots not only exhibit excellent SERS performance with good signals' reproducibility, but also shows the sensitive identification of methyl parathion (one pesticide) on the surface of apples with the detection limit of 10^−8^ mol through the swab sampling approach.

Recently, cotton or polyester swabs are emerging as a new approach to extract analytes, such as explosives, pesticides on fruit surfaces for chemical analysis due to their soft and cost‐effective features.^121^, ^145^, ^146^, ^147^, ^148^ Particularly, Q‐tip cotton‐based SERS swabs have unique advantages. For example, the tip of the cotton swab enables its close contact with the objective surface at the end of tip, which can concentrate and trap the materials, then leading to the magnification of SERS signals.^145^ Furthermore, the Q‐tip cotton based SERS substrates avoid the damage of substrates because of no need to hold the substrate with hands directly.^121^, ^147^ After the self‐assembling of Ag nanoparticles onto the Q‐tip cotton, the lowest detectable amount for 2,4‐dinitrotoluene (2,4‐DNT) can reach ≈1.2 ng cm^−2^ on glass surface, which is 2 orders of sensitive than that of the infrared approach and comparable with that achieved by ion mobility spectrometry‐mass spectrometry. Such cotton‐based SERS swabs hold superior opportunities to be integrated with portable Raman spectrometers to realize numerous onsite chemical detection for POC diagnostics.

## 4 In Situ Detection Based on Flexible SERS

### 4.1 Substrate‐Free SERS for In Situ Detection

The in situ detection strategy based on the SERS technique with rapid and facile features not only affords a unique route for trace identification of specific molecules, such as in live cells or animals, but also provides an effective tool for surface science at the molecular level, including the in situ investigation of solid‐liquid, solid‐gas as well as solid‐solid interfaces and processes.^149^ Such in situ SERS technique has incomparable advantages compared with traditional surface analysis methodologies. For instance, the in situ infrared (IR) spectroscopy can be applied to study active sites or reaction mechanisms, but the detection of intermediates with signals in the low wavenumber region is almost impossible. The basic means via the in situ detection approach is to load the plasmonic nanoparticles straightforward onto the objective surfaces or analytes of interest, followed by the detection of Raman signals.^150^, ^151^ The conventional way is accompanied with rationally preparing bare active nanoparticles (Au, Ag, or Cu) to drive their plasmon resonance peak to well match with the excitation wavelength. Nevertheless, the usage of bare nanoparticles has several intrinsic issues, such as the distortion of vibrational information due to the change of electron density distribution in the molecules or metal catalyzed site reactions. Fortunately, since the discovery of shell‐isolated nanoparticle‐enhanced Raman spectroscopy (SHINERS) pioneered by Tian et al. in 2010, the limitations of bare nanoparticles have been broken.^152^ Such SHINERS technique, regarded as the next generation of advanced spectroscopy, employs a chemically inert shell coating, such as silica, alumina around the metal nanoparticles, which can protect the nanostructures from contact with what is being probed and allow the “smart dust” to conform to arbitrary sample surfaces for the in situ detection.^152^, ^153^, ^154^, ^155^ Meanwhile, the SHINERS technique can help in situ monitor heterogeneous catalytic process to promote the development of more efficient catalysts.^156^, ^157^ However, the SHINERS method directly spreads the active nanoparticles onto the targeted surfaces, which is not easy to control the SERS signals' uniformity for quantitative analyses of chemicals.

**Figure 7:**
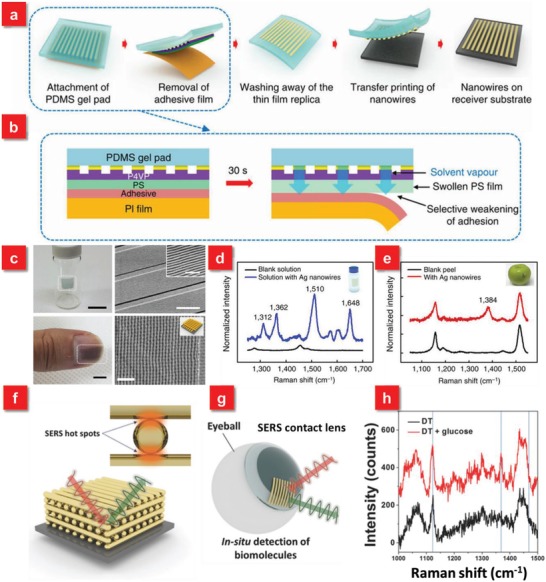
a) Procedure for solvent‐assisted nanotransfer printing. b) The mechanism of transferring the nanostructures and the polymer replica to the surface of PDMS gel pad by removal of the adhesive film. c) S‐nTP of metallic nanowires transferred onto arbitrary surfaces. d) Raman signal spectra obtained from a vial glass containing 10^−5^ m R6G solution. e) Raman signal obtained from the surface of an apple coated with Thiram (tetramethylthiuram disulfide) with an areal density of 1 µg cm^−2^. Raman signal peak at 1384 cm^−1^ corresponds to that of Thiram. Reproduced with permission.^42^ Copyright 2014, Nature Publishing Group. f) A schematic diagram of 3D cross‐point plasmonic nanostructures to enhance SERS performance. g) A schematic illustration of SERS contact lens via transfer printing for in situ detection. h) Comparison of SERS spectra before and after dropping glucose solution, showing the successful detection of glucose. Reproduced with permission.^45^ Copyright 2016, John Wiley & Sons.

To improve the homogeneity as well as increase the hot spots of nanostructures on the objective surfaces for such substrate‐free in situ detection methods, a promising strategy was proposed based on a large‐area sub‐20 nm (down to 9 nm) solvent‐assisted nanotransfer printing (S‐nTP) process.^42^ Via dynamically controlling interface‐specific adhesion using a polydimethylsiloxane gel pad as a solvent‐emitting transfer medium, the ultrafine nanostructures can be uniformly released on diverse receiver surfaces, such as human skin, finger nail, or fruit peels (**Figure** **7**a,b). Compared to conventional nTP methods, which require heat treatment, surface oxidation or liquid bridges to assist the pattern transfer, the S‐nTP approach relies on the injection of solvent molecules from the wet PDMS gel pad to facilitate the release of ultrahigh‐resolution nanostructures onto almost arbitrary receiver surfaces (Figure 7c,d). Such SERS substrates based on the transfer‐printing approach exhibits high uniformity of Raman signals over a large area (≈1 cm^2^) with a standard deviation of 5–9%. Meanwhile, the detection limit of Thiram, a widely used pesticide, on the apple surface can be down to 1 µg cm^−2^ after the attachment of plasmonic nanostructures, which is lower than the maximally permissible pesticide residue level for fruits (22–31 µg cm^−2^) (Figure 7e). Furthermore, in order to simplify the S‐nTP process as well as further boost the SERS performance for in situ detection, the same group developed the second‐generation S‐nTP to generate multilayer stacks of nanowires.^45^ The big difference is that the new S‐nTP method employs a single‐layer polymethylmethacrylate (PMMA) replica as a high‐yield transfer medium, eliminating the need of an additional transfer medium (previously PDMS gel) and a transfer step. The fabricated 3D cross‐point plasmonic nanostructures over a large uniform area demonstrate a prominent enhancement of Raman signals with an average enhancement of 4.1 × 10^7^, which is attributed to the in‐plane coupling effect in the nanogap region and out‐of‐coupling effect at the cross‐points (Figure 7f). The 3D flexible SERS substrate based on the novel S‐nTP approach is served as a substrate‐free “SERS contact lens,” which can be used for in situ detection of glucose levels in tears to monitor diabetes (Figure 7g,h).

### 4.2 Flexible Material–Based SERS for In Situ Detection

**Figure 8:**
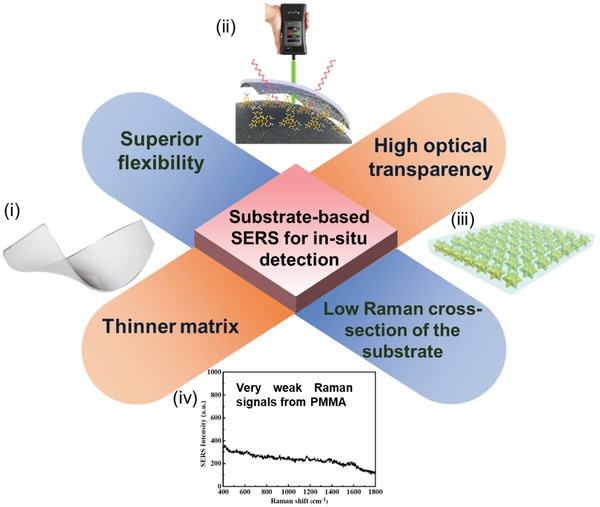
The crucial features of substrate‐based SERS for in situ detection. i‐ii) Reproduced with permission.^40^ Copyright 2017, American Chemical Society. iii) Reproduced with permission.^159^ Copyright 2017, American Chemical Society. iv) Reproduced with permission.^160^ Copyright 2014, American Chemical Society.

Besides the utilization of substrate‐free nanostructures for in situ SERS detection, it is highly essential to develop the hybrid plasmonic nanostructures on the supporting substrates with high flexibility, which can increase the reproducibility as well as the operability of the in situ SERS approach for POC applications. Until now, plenty of flexible substrates have been applied for in situ SERS detection, including PDMS,^69^, ^78^, ^158^, ^159^ PMMA,^79^, ^160^, ^161^, ^162^ polyethylene terephthalate (PET),^163^, ^164^, ^165^ polyethylene (PE),^166^ poly(vinylpyrrolidone) (PVP),^82^ poly(ε‐caprolactone) (PCL),^40^ adhesive tape,^74^, ^167^ as well as nanowires.^43^, ^74^, ^168^, ^169^ As shown in **Figure** **8**, different from the aforementioned swab‐sampling approach primarily highlighting the flexibility as its pivotal feature, the in situ SERS strategy requires not only more superior flexibility and ductility but also high transparency of the substrates because of the demand to excite incident photons and then collect Raman signals from the back side of the SERS substrates. Furthermore, the thickness of the substrate should also be taken into considerations for those complex surfaces, where the thinner matrix is typically preferred. In addition, because of the requirement to excite laser from the opposite side of SERS substrates in the in situ detection, the Raman signals from the supporting substrate should be inhibited. These unique characteristics of flexible substrates promote their intimately conformal contact with the arbitrary objects for in situ detection in diverse applications.

**Figure 9:**
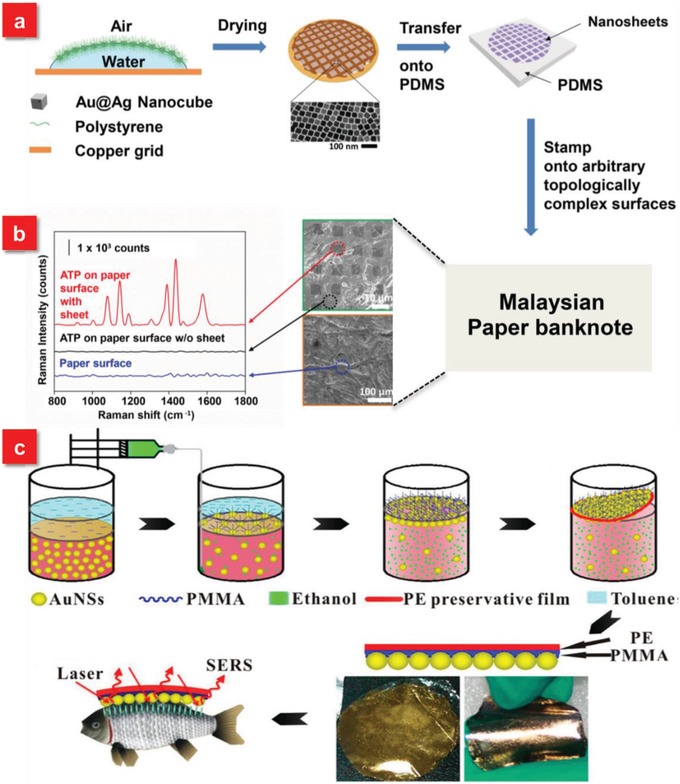
a) Scheme for fabrication process of nanocube‐plasmene nanosheet via a polystyrene‐based drying‐mediated self‐assembly and PDMS‐mediated transfer for utilization as SERS adhesive. b) 4‐ATP Raman spectra and SEM images of plasmene nanosheet on topologically complex surfaces of Malaysian paper banknote. Reproduced with permission.^170^ Copyright 2015, John Wiley & Sons. c) Schematic representation of preparation of AuNPs/PMMA SERS substrate and its application for in situ detection of MG on fish surfaces. Reproduced with permission.^160^ Copyright 2014, American Chemical Society.

Among the numerous flexible materials, PDMS is one of the most widely used materials for in situ SERS detection, which is a silicon‐based organic polymer with highly transparent, inert and nontoxic properties. The thickness of the PDMS membrane can be controlled down to several micrometers depending on the depth of the used molds or the spin speed. The flexibility, softness as well as optical transparency of the PDMS membrane fabricated with active nanostructures allows them to be attached onto the different surfaces of interest.^78^, ^158^, ^170^, ^171^ Based on these superior features, Chen et al. demonstrated a new‐class of soft plasmene nanosheets with Au@Ag nanocubes as the building blocks using a facile yet efficient bottom‐up self‐assembly approach (**Figure** **9**a).^170^ The high mechanical flexibility of the nanosheets enables them to be transferred to the PDMS elastomer, which is used as a powerful tool for trace amount of chemicals on Malaysian banknote (fibrous), Australian banknote (polymer) as well as Australian coin (rough metal) for in situ diagnostics (Figure 9b). Similar to PDMS, the newborn PMMA is also a very fashionable material to be served as a template for flexible in situ detection due to its excellent optical transparency (Figure 9c).^160^ Recently, a new type of flexible and self‐standing silicon nanowire networks was reported for the in situ and nondestructive sensing. The abundant hot spots can be formed because of the electric field enhancement from the individual nanowires and electromagnetic coupling between the closely separated nanowires, leading to the in situ detection of pesticide residues on the fruit surface with the detectable concentration down to 72 ng cm^−2^.^43^

**Figure 10:**
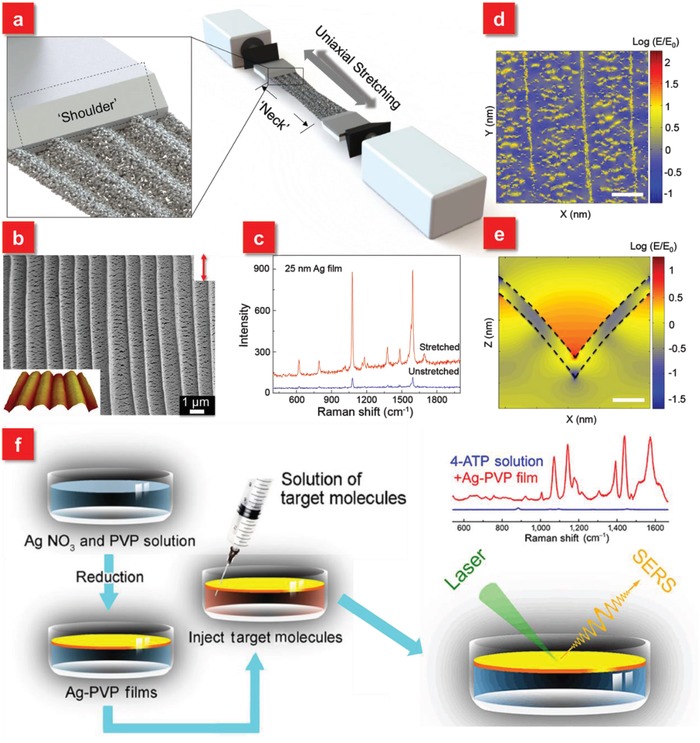
a) Schematic diagram of stretching polymer SPR film under an external mechanic force. b) SEM image of stretched polymer SPR film. The inset shows AFM image of four complete periods of polymer SPR film with 3D wave‐shaped structures c) SERS spectra of 4‐MBT molecules adsorbed on polymer SPR film before and after the stretching. Calculated electric field distributions of the d) microribbons (scale bar: 500 nm) (*x*–*y* plane) and e) nanogrooves (scale bar: 50 nm) (*x*–*z* plane). The thickness of simulated Ag film is 25 nm. Reproduced with permission.^40^ Copyright 2017, American Chemical Society. f) Schematic diagram of the designed Ag‐PVP films for in situ SERS detection on the liquid phase. The comparison of Raman spectra of 4‐ATP solution with and without Ag‐PVP films shown in the top right corner. Reproduced with permission.^82^ Copyright 2016, Springer Nature.

The traditional approaches to fabricate active nanostructures on the transparent and flexible SERS substrates rely on the deposition of nanoparticles by physical or chemical routes. Particularly, the realization of homogeneous nanostructures requires the state‐of‐art lithographic techniques, followed by the pattern transfer onto the flexible substrates. It is thus essential to develop a facile, yet efficient approach to generate uniform nanostructures on the flexible substrates over a large area. Furthermore, the most general single‐use SERS substrates involve the production of environmentally unfriendly by‐products and waste streams, which violate the goal of environment protection.^172^ In view of this issue, our group developed a new biocompatible and biodegradable SERS substrates via irreversibly and uniaxially stretching metal deposited flexible PCL SPR film as wearable sensors for in situ detection of analytes.^40^, ^173^ The PCL film, as an excellent flexible, biodegradable and biocompatible material with good transparency (≈90%) and temperature stability (9.62%), is for the first time employed as a building block for flexible SERS substrates. After the deposition of Ag film, the composite film after stretching exhibits surprising phenomena: 3D and periodic wave‐shaped microribbons array embedded with a high density of nanogaps functioning as hot spots at an average gap size of 20 nm and nanogrooves array along the stretching direction (**Figure** **10**a,b). The stretched polymer surface plasmon resonance film gives rise to more than ten times signal enhancement in comparison with that of the unstretched composite film due to the localized fields within the nanogaps and nanogrooves (Figure 10c–e). Furthermore, the ultrathin property (≈10 µm) of the SPR film is expected to access small corners of complex surface, such as carambola, which is very easy to hide and stay with residual pesticides. Following the approach of uniaxial stretching, bi‐axially stretching of polycarbonate (PC) film, a thermoplastic material, is recently demonstrated by Li et al., which also allows the generation of nanostructures with good controllability, leading to the high‐performance SERS film for in situ sensing.^174^ Such stretched‐induced homogeneous nanostructures can be an alternative route to produce high‐throughput SERS active substrates for immediate use in case of the chemical contamination.

The aforementioned applications make use of the flexible and transparent SERS substrates to be conformally wrapped on the surfaces of solid‐phase substrates for in situ detection, while few studies focus on the flexible SERS on a liquid phase for the direct detection of analytes, although this is also an important field. For instance, aquatic environments have been seriously contaminated by multiple organic pollutants and toxic metal ions etc. due to the increased agricultural and industrial activities. The detection of water quality is thus highly essential. However, the traditional manual sampling processes and data analyses are too complex. Hence, it is desirable to develop a universal SERS platform with onsite characteristics for real‐time monitoring in aqueous solution.^175^, ^176^ With this motivation in mind, Wang et al. demonstrated a facile fabrication approach by encasing Ag particles in PVP film (Ag‐PVP films) at the gas–liquid interface through an one‐step electronic reduction procedure (Figure 10f). Such Ag‐PVP films are capable of floating on top of water, allowing to in situ detect various molecules in a liquid phase.^82^ Furthermore, a green and reusable SERS substrate composed of PMMA/Ag NPs/graphene has been realized for in situ detection of Thiram in apple juice. The good transparency of the substrate enables the incident light to pass through the flexible substrate and reach the hot spots to amplify SERS signals.^177^ Such versatile flexible SERS substrates have unique advantages of directly monitoring the analytes in liquid phases through coupling with a handheld Raman spectroscope without tedious sampling procedures for POC and in‐field analyses.^82^, ^177^, ^178^ **Table** **1** summarizes the aforementioned three categories of flexible SERS platforms in terms of primary backbone materials, plasmonic compositions, fabrication methods, unique features, applications as well as detection limit for each flexible SERS substrate.

**Table 1:** Summary of the three catagories of flexible SERS substrates

Types of flexible SERS platforms	Backbone materials	Compositions	Fabrication methods	Unique features including flexibility	Applications	Detection limit	Ref.
Actively tunable SERS	PDMS	Au NPs on colloidal particle arrays	Nanosphere lithography, soft lithography	Tunable plasmonic extinction ranging from 581 to 625 nm	Real‐time optical tunable sensors	10 × 10^−9^ m (BT)	Kang et al.^101^
	PDMS	Ag nanoislands	Metal film dewetting and nanoisland transfer	Tuning resolution of 1 nm	A guideline to engineer SERS substrates		Jeong et al.^75^
	Hydrogel	Au NPs	Cast method	An open‐to‐closed system	Bio‐macromolecules' detection		Mitomo et al.^76^
	PDMS	Ag nanorods array	Oblique angle deposition	Strain induced wrinkles	Bacterial detection	1 × 10^4^ CFU mL^−1^ (P. aeruginosa.)	Kumar et al.^47^
	PDMS	Wrinkled graphene/Au NPs	Graphene transfer/physical deposition	50% tensile strain without performance degradation	Multiple analytes' detection	10^−9^ m (R6G)	Chen et al.^70^
Swab‐sampling approach	Glass‐fiber filter paper	Ag NWs	Mixing and vacuum filtration	High capture capability of pesticides	Onsite residual pesticide detection	40.2 ng cm^−2^ (PQ)	Koh et al.^169^
	Bacterial nanocellulose	Au NRs	Gravity‐assisted filtration method	3D porous structures, biocompatible	Bacterial recognition	10^−9^ m (R6G)	Tian et al.^128^
	PDMS	Ag NRs	Glancing angle deposition	Excellent mechanical stability	Pesticide residues' detection	10^−9^ g cm^−2^ (Thiram)	Kumar et al.^138^
	Commercial tape	Au NPs	Drop‐dry method	“sticky” feature	Pesticide residues' detection	0.24 ng cm^−2^ (Thiram)	Chen et al.^41^
	Cotton	Ag NPs	Self‐assembly	Easy to operate (Q‐tip)	Explosive detection	1.2 ng cm^−2^ (2, 4‐DNT)	Gong et al.^147^
	Copper net	Ag nanoflowers/graphene	CVD/Electrodeposition	Graphene nanogap to provide hot spots	Pesticide residues' detection	10^−14^ m (R6G)	Zhang et al.^124^
In situ detection for SERS	PCL film	Ag film	Uniaxially stretching	3D wave‐shaped microribbons array embedded with nanogaps	Environmental monitoring	0.1 × 10^−6^ m (MG)	Xu et al.^40^
	Substrate ‐free	Ag/Au nanowires	Nanotransfer printing	3D cross‐point nanostructures	Glucose detection	10^−11^ m (R6G)	Jeong et al.^45^
	PMMA	Graphene/Ag ‐nanoflowers	Chemical reduction method	Long‐term stability	Food security	10^−14^ m (R6G)	Qiu et al.^79^
	Scotch tape	Silica nanowires/Ag NPs	Bottom‐up route	Free‐standing 3D networks	Pesticide residues' detection	10^−9^ m (4‐MPY)	Liu et al.^74^
	PVP	Ag NPs	One‐step electronic reduction	Floating metal film	Liquid‐phase detection	10^−11^ m (4‐ATP)	Wang et al.^82^

## 5 Other Emerging Flexible SERS Platforms for Novel Applications

**Figure 11:**
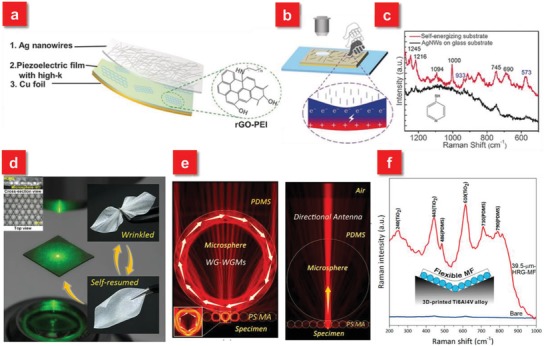
a) Schematic illustration of the E‐SERS active device. b) Schematic illustration of the finger‐press‐promoted E‐SERS: electrical energy was generated and stored in the film deformation process. c) The Raman spectra of 4‐MP collected from the apple surface using the self‐energizing substrate and AgNWs on a glass substrate. Reproduced with permission.^83^ Copyright 2017, John Wiley & Sons. d) Real image of backscattering configuration for microsphere‐enhanced Raman spectroscopy (MERS). The wrinkled and self‐resumed microsphere‐embedded film exhibits its excellent flexibility. Top and cross‐section views of a 65 µm BaTiO_3_ glass microsphere film shown in the left right corner. e) Wave‐guided whispering‐gallery modes and direction antenna effect to enhance Raman scattering. f) MF‐enhanced Raman detection of TiO*_x_* on a 3D‐printed Ti6Al4V alloy lightweight component by rapid mapping without excitation laser focusing. Reproduced with permission.^181^ Copyright 2017, American Chemical Society.

In addition to the above mostly reported three categories of flexible SERS substrates, several new classes of flexible SERS platforms recently spring up, such as electrochemically SERS (E‐SERS) based on energy conversion,^83^ “pen‐on‐paper” approach,^179^, ^180^ microsphere‐enhanced Raman spectroscopy,^181^, ^182^ as well as anticounterfeiting security labels.^80^, ^183^ These emerging SERS techniques push forward their practical and diverse applications as flexible wearable sensors. For instance, a flexible self‐energizing SERS substrate is demonstrated via integrating the active nanostructures with energy conversion and storage all‐solid‐state flexible film materials (**Figure** **11**a).^83^ The SERS signals can be enhanced up to ten times by simply pressing the self‐energizing substrate with a finger. The distinct trait of this flexible substrate is that the use of piezoelectric polymer film as a matrix coupled with surface engineered reduced graphene oxide (rGO), both providing high permittivity as well as low dielectric losses, which are able to convert the film deformation into stored electrical energy (Figure 11b). Such self‐energizing SERS substrate with high flexibility demonstrates potential opportunities in fast onsite detection. By slightly rubbing the flexible SERS substrate on the apple surface, the electrical potential is generated, simultaneously allowing the transfer of pesticide residue from the apple surface to the flexible SERS substrate, which not only provides the enhancement of SERS signals but also affords the route in potable on‐spot applications (Figure 11c).

In recent years, an emerging approach called “microsphere‐enhanced Raman spectroscopy” is introduced to enhance Raman signals. Due to the excellent optical properties of dielectric microspheres, such as photonic nanojets, whispering‐gallery modes (WGMs) and directional antennas effect, microspheres are able to manipulate light focusing, confinement as well as scattering on the microscale, which can be flexibly coupled with SERS active nanostructures to provide an extra enhancement.^181^, ^182^, ^184^, ^185^, ^186^, ^187^ As such, several groups have demonstrated the enhancement of Raman scattering signals mediated by photonic nanojet, which is capable of tremendously boosting the localized near field with several orders. However, the most of microsphere‐enhanced Raman spectroscopy methods require the microsphere suspension dropped on the specimen surfaces. The samples are thereby destroyed and more importantly, the position of microsphere with respect to the hot spots is hard to be controlled. To solve these problems, Xing et al. demonstrated a flexible dielectric microsphere‐embedded film with a transparent PDMS as the matrix (Figure 11d).^181^ Due to the focusing properties of the microsphere, the enhancement ratio of Raman intensity can be increased at least one order of magnitude (Figure 11e). Furthermore, because of its excellent flexible behavior as well as user‐friendly nature, the microsphere‐embedded film can serve as a sensing platform, which can be used for enhancing Raman signals of a variety of specimens ranging from 1D to 3D (Figure 11f).

**Figure 12:**
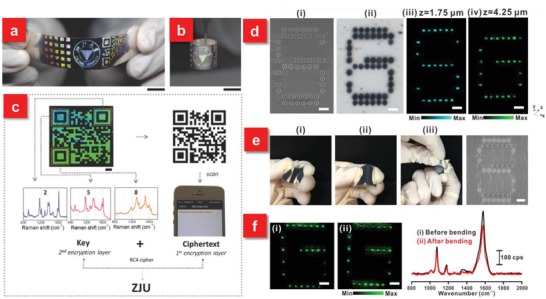
a) Coding encryption capability of the flexible plasmonic metafilm with a 5 × 5 iridescent square array, the logo of Zhejiang University, and a quick response (QR) code. b) Demonstration of the flexibility of the plasmonic tape. c) Example of an encoding strategy using the iridescent QR system. Scale bars: a,b) 1 cm; c) 2 mm. Reproduced with permission.^80^ Copyright 2016, John Wiley & Sons. d) Demonstration of a flexible 3D SERS security label. e) Such security label subjected to bending, flexing, and twisting forces. f) SERS images and the corresponding SERS spectra of the letter “G” i) before and ii) after 50 mechanical stress cycles. All scale bars represent 5 µm. Reproduced with permission.^183^ Copyright 2017, American Chemical Society.

The aforementioned flexible SERS substrates primarily concentrate in discussing the state‐of‐the‐art SERS techniques for biosensing in environmental monitoring, food safety diagnostics drug detection and healthcare inspection. On top of that, the smart SERS technique, especially building from the flexible platforms, affords significant potentials in anticounterfeiting applications. As a global longstanding problem, counterfeiting and forgery have caused negative impacts on the national economy and security. Among various strategies to address this issue, SERS is considered as a promising one, which can convert the molecular information on reporter molecules adsorbed on plasmonic nanostructures to SERS imaging. Different from the anticounterfeiting technologies based on complex plasmonic metasurfaces together with manipulating their optical properties or fluorescent encoding, requiring multiple processing to resolve overlapped spectra, the SERS imaging approach relies on narrow Raman fingerprints of molecules, which are embedded in the anticounterfeiting systems and are not visible to the public.^188^ Several groups have made huge efforts toward the investigation of SERS‐based anticounterfeiting using a couple of effective methods, such as polarization‐dependent responses,^189^, ^190^, ^191^ multiple probe molecules embedded in the plasmonic nanostructures,^192^, ^193^ and 3D candlestick microstructures to extend the encoding capacity.^183^ Particularly, the rational use of flexible substrates with superior mechanical flexibility and robustness breaks the limitations of current rigid plasmonic security labels (**Figure** **12**). The integration of anticounterfeiting systems with the flexible platforms holds a great opportunity as an advanced next‐generation anticounterfeiting security label, which can be conformally attached onto arbitrary irregular objects, such as banknotes, documents and medicine vials for future anticounterfeiting security solutions.

## 6 Conclusions and Outlook

In the past few years, the rising interest in the research field of flexible SERS sensing has stimulated the advances of SERS technique from a laboratory‐based tool extended to practical real‐world applications. Although tremendous research efforts have been involved in the rational design of active uniform nanostructures on rigid matrices with a high density of hot spots over a large area, their intrinsic limitations make the SERS technology primarily play roles in the laboratory settings' environment. In order to satisfy the requirement of increasingly demanded POC diagnostics for onsite and real‐time monitoring, the emerging flexible SERS devices have attracted unprecedented research attentions. Such flexible SERS techniques are not only endowed with the capabilities that the rigid substrate–based SERS chips have, but also can provide distinct features and functionalities to fulfill real‐life tasks. In this progress report, we critically present three main classes of the flexible SERS sensors in depth. The unique advantages as well as crucial issues of the flexible SERS devices are highlighted. Based on the practical applications that the flexible SERS devices aim to be used in, a variety of flexible materials are employed and compared.

However, in order to completely transform the flexible SERS technique into real‐life applications for effective and accurate POC diagnostics, a plurality of challenges still remain. For example, the stretchability of flexible materials makes it possible to actively control the position of plasmon resonance peak associated with the excitation wavelength to achieve the optimum SERS performance. Nevertheless, the resonance tunable range is relatively narrow, which limits their practical applications. More research is thus encouraged to be invested in exploring the broadband tunable capability of plasmon resonance. Meanwhile, regarding the swab sampling–based SERS applications, the adhesion among the flexible substrate, active nanostructures and analytes on the objective surfaces should be further considered and improved, which will greatly influence the intensity of Raman signals. Furthermore, current studies primarily focus on the versatile detection of pesticides or pollutants for environmental monitoring and food safety evaluation by means of flexible SERS platforms. The analytes are usually well‐known Raman reporters. However, there is few research concentrating in investigating the multifunctional flexible SERS devices in bio‐applications for healthcare diagnostics. As the common symptoms (e.g., fever, cough) are mostly associated with several infectious sources, such as viruses or bacteria, it is of high significance to be capable of precisely performing multiplexed identification, which could be probably addressed by integrating the flexible SERS platforms with microfluidic technologies. Meanwhile, the Raman signals between normal and cancerous cells are usually similar. Statistical analyses methods have been applied to distinguish variations in a dataset, but their reliability should be further improved. In addition, the current flexible SERS substrates are usually built from polymer materials with relatively strong background Raman signals from the supporting matrices, especially at the higher intensity of laser excitation. The exploration of new flexible substrates with intrinsic low Raman signals is also crucial for POC testing.

For the practical real‐world applications, the cost should also be considered in POC settings, including the materials' and fabrication's cost. In case of contamination, the SERS chips are usually for single use. The current high‐performance SERS substrates with superior sensitivity are principally based on noble metal–based nanostructures, the high cost of which hinders their large‐scale production for practical applications. It is greatly essential and urgent to find novel materials with high‐performance SERS effect, although several semiconductor materials have been reported with moderate SERS phenomena.^194^, ^195^, ^196^, ^197^ As for the fabrication cost, new facile yet efficient approaches should be exploited to prepare homogeneous plasmonic nanostructures with intensive SERS signals on the flexible substrates, such as the stretching approach,^40^, ^174^ laser micro/nanoprocessing,^198^, ^199^, ^200^, ^201^, ^202^, ^203^ and pattern transfer from natural templates.^204^, ^205^, ^206^ The development of recyclable and flexible SERS substrates is also an alternative strategy to realize cost‐effective practical applications. Because of their self‐cleaning characteristics, the SERS substrates are capable of being reused for multiple times almost without the performance degradation by an external stimulus.^207^, ^208^, ^209^, ^210^ Furthermore, to make the onsite and real‐time POC diagnostics detection come true, the development of portable Raman spectroscope should not be lagged. The current portable Raman spectroscopy on the market is relatively expensive (>20 000 $), which is unfavorable to penetrate into households. The size of device should be further decreased with higher sensitivity. In addition, to satisfy the POC testing in different fields, multiple excitation laser wavelengths from the visible to near infrared range should be covered and data libraries should be enriched in the portable Raman system.

Furthermore, to comprehensively diagnose the health status of individuals, the development of flexible SERS technique alone is insufficient. Other flexible physical/chemical sensors should be exploited as well to satisfy the increasing demand of emerging Internet of Things (IoTs). Fortunately, a great deal of efforts in the past decade have been devoted to exploring intelligent wearable sensors to monitor human physiological information, such as body temperature, pulse rate, respiratory rhythm, blood pressure and sweat etc.,^211^, ^212^, ^213^, ^214^, ^215^ although the performance of these flexible devices should be further improved for practical use in terms of sensitivity, response/ recovery time, portability as well as superior reliability. Meanwhile, the study of human‐interactive devices has been transited from “one system = one result” to “one system = several results” so as to synchronously acquire diverse vital signs.^19^, ^216^, ^217^ The portable flexible SERS sensor is also envisioned to be integrated into these multifunctional wearable sensor systems to play its unique complementary role as the next‐generation POC diagnostics in a variety of applications, especially in resource‐limited settings.

## Conflict of Interest

The authors declare no conflict of interest.
